# A novel methodology for the optimization of transmission and dosimetric leaf gap parameters

**DOI:** 10.1002/acm2.13565

**Published:** 2022-02-28

**Authors:** Dominic J. DiCostanzo, Ahmet S. Ayan

**Affiliations:** ^1^ The Ohio State University Columbus Ohio USA

**Keywords:** commissioning, dosimetric leaf gap, multileaf collimators, quality assurance, treatment planning systems

## Abstract

**Purpose:**

Optimization of dosimetric leaf gap (DLG) and transmission is commonly performed through a manual trial and error process, which can lead to sub‐optimal values. The purpose of this work is to create an alternative automated optimization process that provides the optimal DLG and transmission pair for use in a clinical setting.

**Methods:**

Utilizing the treatment planning system application programming interface, a phase space of clinically viable DLG and transmission pairs was generated. The phase space contained 51,051 dose planes for DLGs between 0.0  and 2.5 mm and transmission values between 0.01% and 2.5%. Thirteen plans were measured for multiple multileaf collimator types and nominal beam energies. The optimization minimized the mean *γ*‐index and maximized the *γ*‐index pass rate. The optimized values were validated using five plans excluded from the optimization.

**Results:**

Of the nominal beam energies and multileaf collimator system (MLC)‐type combinations tested, 6/7 showed an increase in *γ*‐index pass rate and a decrease in mean *γ*‐index signifying better agreement between measurement and calculation. When comparing the optimized DLG and transmission values to the clinically implemented values identified via an iterative method, 5/7 energy, and MLC type combinations showed no statistically significant changes. In addition, the optimized values were benchmarked against three Task Group 119 plans with published *γ*‐index pass rates, which had been held out of the optimization. For those plans, the optimized DLG and transmission values provided the same or better *γ*‐index pass rates.

**Conclusion:**

We presented a novel and viable automated alternative to current approaches of selecting the DLG and transmission parameters. This method will reduce the time required to determine the clinically acceptable DLG and transmission parameters and ensure optimality for the plans included in the optimization.

## 1 INTRODUCTION

Multileaf collimator systems (MLCs) have become a standard accessory in the delivery of conformal and modulated radiation therapy. Each linear accelerator vendor has implemented MLCs differently^1^ presenting unique challenges to modeling in the treatment planning system (TPS). The Millennium MLC system developed by Varian Medical Systems (Palo Alto, CA) has two main configurations available: the 120‐leaf (SD) and HD120‐leaf (HD). Both MLC systems are single‐focused, use a tongue‐and‐groove design, and have rounded leaf ends. The combination of the rounded leaf ends, and the modeling approach implemented in the Eclipse TPS complicate the beam modeling and commissioning process. Specifically, the availability of only two adjustable parameters to achieve optimal matches between calculation and measurement for modulated plans: dosimetric leaf gap (DLG) and transmission. Compounding this challenge, both the HD and SD‐MLC systems include multiple widths of MLCs, and as a result, exhibit different DLG and transmission values in different locations of the field.^2^, ^3^

Both DLG and transmission are physical attributes of the MLC system that are a function of individual machine characteristics and nominal beam energies. LoSasso et al.^4^ is often credited with being the first to develop a method of measurement for the DLG parameter by using a sweeping gap technique. While the DLG and transmission parameters are physical in nature, the implementation of the dose algorithms in the TPS requires these values to be adjusted to provide the best match between calculation to measurement.

There have been multiple efforts to simplify and optimize the measurement and adjustment of the DLG and transmission parameters over the years.^5^, ^6^, ^7^, ^8^, ^9^, ^10^, ^11^, ^12^, ^13^, ^14^ Chauvet et al.^5^ characterized dosimetric aspects of the transmission and the dosimetric leaf separation factor (more commonly, DLG) by using a sliding slit test. Within the study, the DLG was held constant, and transmission varied, and conversely, the transmission was held constant with varied DLG. Of note, it was identified that there exist multiple values of DLG and transmission for a given plan that provide an agreement between calculation and measurement.

Recently there have been empirical and analytical approaches to optimizing the DLG and transmission values. While some have developed specific test fields,^9^, ^11^, ^14^ Xue et al.^13^ provided a novel method for optimizing volumetric modulated arc therapy (VMAT) plans using *γ*‐pass rates. By varying DLG and calculating the associated *γ*‐pass rate for multiple plans, polynomials were fit and solved to determine the optimal DLG value for each plan. However, the transmission value was held constant when others^5^, ^14^ have shown the interaction of DLG with the transmission. Additionally, the number of plans used during the optimization was small and the sampling of the DLG was coarse.

The aim of this work was to develop a method to optimize the DLG and transmission parameters using a phase space of clinically relevant treatment plans that were automated and agnostic to MLC type, nominal beam energy, and dose algorithm. By utilizing a precalculated phase space of clinical treatment plans, the commissioning process could be streamlined and made robust against suboptimal results. We hypothesized that the method developed would achieve similar results to parameters implemented clinically, which were commissioned using an iterative approach.

## 2 METHODS

**FIGURE 1:**
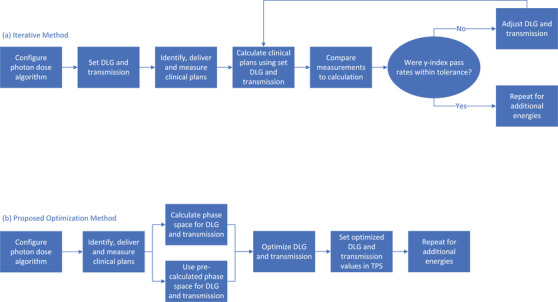
Pane (a) displays the commonly used workflow where iterative adjustments are made to the dosimetric leaf gap (DLG) and transmission after manual comparison to the *γ*‐index pass rates of multiple plans. Pane (b) displays the workflow proposed using this methodology whereby the optimization process identifies the DLG and transmission values using either a pre‐calculated phase space or via a newly created phase space

To achieve our aim, we leveraged the TPS application programming interface, Eclipse scripting API, to calculate pairs of DLG and transmission values spanning the clinically relevant range for all MLC types and nominal beam energies in our institution. The unaltered clinical plans were delivered and measured using a two‐dimensional (2D) diode array. Using the results of the γ‐index^15^ analysis, we identified a cost function based upon the mean γ‐index and optimized DLG and transmission simultaneously for a set of clinical treatment plans. Further, we validated these results on plans omitted from the optimization process. A comparison of our method to the iterative method used in most clinics is shown in Figure 1.

### 2.1 DLG/transmission phase space

Two beam models were created for two models of MLC (SD and HD). The TrueBeam Representative Beam Data for Eclipse was used as the basic beam data for the models of each energy and the Anisotropic Analytical Algorithm version 15.6.0.5 was commissioned. The target spot size was set to 1.0 mm in the x‐direction and 0 mm in the y‐direction as suggested in the algorithms reference guide.^16^ No other modifications were made to the available parameters after the automatic configuration of the beam model. The MLC transmission values were varied from 0.01% to 2.5% in 0.25% increments for photon beam energies of 6X, 6X‐FFF, 10X, 10X‐FFF, and 15X. For each transmission value, the dose was calculated for DLG values ranging from 0 to 2.5 mm in 0.05 mm increments.

The DLG is used by the TPS during dose calculation whereby each MLC leaf is shifted by one‐half of the entered DLG value.^16^ However, the DLG parameter is not accessible programmatically. As a result, the DLG in the TPS was held constant at the minimum allowable value, 0.001 mm. To achieve DLG values in lieu of manually setting the static values in the beam model configuration, each MLC leaf was physically moved by one‐half the aforementioned values (i.e., for a DLG value of 1.25 mm each leaf position was adjusted by 0.625 mm). Unlike the DLG, an automated adjustment of the transmission parameter is not possible. As a result, the transmission parameter was manually adjusted prior to calculating each plan for the specified DLG values.

The resultant DLG and transmission phase space consisted of 561 treatment plans (11 transmission values, and 51 DLG values for each transmission value) for each of the 13 selected plans for optimization for each nominal beam energy (see Table 1 for a sample of the plans used). Two plans used were standard intensity‐modulated radiotherapy (IMRT) commissioning plans: Fluence and Chair. The Fluence plan is a required plan for portal dosimetry commissioning that implements variable‐sized delivered fluences without jaw tracking in order to determine the kernels for the Portal Dose Image Prediction Algorithm.^16^ The Chair pattern was developed by Van Esch et al. and has three regions.^11^ Each of these regions provides unique information regarding DLG and transmission. Additionally, five plans from the American Association of Physicists in Medicine Task Group 119 (TG119) were calculated but omitted from the optimization to use during validation.^17^ The total number of plans generated for all energies and MLC combinations was 51,051 and 19,635 for optimization and validation, respectively.

**TABLE 1:** Sample of plans used for optimization and validation for HD‐MLC and 6X energy. Other combinations of multileaf collimator systems (MLCs) and energies used the same plans, the same number of fields, same collimator angles, with different monitor units (MU) in some cases

**Phase used**	**Plan id**	**Number of fields**	**Field collimator angles [degrees]**	**Jaw tracking enabled**	**Bounding box of 50% isodose line at measurement plane (width x height, cm^2^)**	**Prescribed dose [cGy]**	**Monitor units [MU]**
Optimization	ABD	6	0	N	14.4 × 13.9	200	894
Optimization	BRN	7	0	N	12.4 × 11.6	200	1431
Optimization	Chair	1	0	N	14.3 × 15.2	100	200
Optimization	CW	8	15 and 345	N	22.7 × 19.0	200	2090
Optimization	DLG	1	90	N	13.5 × 18.4	100	200
Optimization	Fluence	1	90	N	20.7 × 12.3	100	199
Optimization	HN	7	0	N	8.5 × 19.9	200	980
Optimization	*HNSS	7	0	Y	8.5 × 19.9	200	503
Optimization	PLV	9	90	N	17.3 × 19.0	200	2582
Optimization	HNV1	9	0	Y	15.0 × 19.5	212	2618
Optimization	HNV2	9	0	Y	17.4 × 20.4	200	2870
Optimization	HNV3	9	90	Y	19.8 × 20.6	212	4493
Optimization	HNV4	9	90	Y	17.6 × 23.0	200	6167
Validation	HC	9	0	N	8.7 × 9.2	200	2013
Validation	SC	9	0	N	8.3 × 10.0	200	1148
Validation	SWL	1	0	N	13.5 × 5.1	200	972
Validation	SWR	1	0	N	21.2 × 20.5	200	1007
Validation	MT	7	0	N	22.0 × 15.0	200	651

* HNSS was the only plan tested that was delivered with step‐and‐shoot delivery. All other plans were delivered using a sliding window technique.

The three‐dimensional dose distribution for each plan was calculated in a water phantom. For each calculated plan, a 2D dose plane at 5‐cm depth was then exported corresponding to the isocenter location and measurement plane. A computer program (written in C#) was written to automate the process of exporting the calculated 2D dose distributions as Digital Imaging and Communications in Medicine (DICOM) radiotherapy (RT) dose files using Evil DICOM.^18^, ^19^

### 2.2 Delivery and measurement of plans

Each of the original and unaltered treatment plans was exported as DICOM RT Plan files and stored in a location that was accessible to the treatment machines.^18^ Varian TrueBeam machines were used to deliver the plans in Quality Assurance mode. One machine was equipped with HD‐MLCs and nominal energies of 6X, 6X‐FFF, and 10X‐FFF, and the other was equipped with SD‐MLCs and nominal energies of 6X, 6X‐FFF, 10X, and 15X. A MapCHECK2 with MapPHAN (SunNuclear Corporation, Melbourne, FL) diode array was used to measure the delivered plans. SNC Patient (SunNuclear Corporation, Melbourne, FL) was used to perform an array and dose calibration prior to measurements, and to acquire the data.

### 2.3 *γ*‐index calculation

The comparison between calculated and measured 2D dose distributions was done using the *γ*‐index.^15^ A MATLAB (MathWorks Natick, MA) computer program was developed to calculate the 2D *γ*‐index. The accuracy of the developed *γ*‐index calculation code was bench‐marked using the test dose distributions provided in Agnew and McGarry.^20^ The software was written to allow expedient calculation of the *γ*‐index for each plan in the phase space as compared to measurement.

### 2.4 Optimization of DLG and transmission parameters

To begin, both *γ*‐index pass rates and mean *γ*‐index were calculated for all plans. The criteria used was a 10% dose threshold with the DTA and %DD criterion of [2 mm, 2%] using global normalization. The pass rate was determined by taking the percentage of measured pixels with *γ*‐value less than or equal to unity divided by all measured pixels above the dose threshold. Additionally, the mean *γ*‐index was calculated by summing all *γ*‐values for each pixel and dividing by the number of pixels above the dose threshold.

A MATLAB program using the *fmincon* ^21^ routine optimized the DLG and transmission parameters using separate cost functions for each pair of nominal beam energy and MLC type (*N* = 7). Two cost functions were evaluated: the sum of the pass rates (*F*1) and the sum of the mean of *γ*‐index values per plan (*F*2) for all plans:

$$F1\;=\;max\left\{\sum_{i\;=\;1}^{allplans}{\left[\Gamma PassRate\right]}_{i}\right\},$$

and

$$F2\;=\;min\left\{\sum_{i\;=\;1}^{allplans}{\bar{\Gamma }}_{i}\right\}.$$

The optimization routine maximized *F*1 and minimized *F*2. For each pair of energy and MLC type, the optimization was repeated with different starting values to ensure stability and reproducibility of the optimization. A spline interpolation was used to calculate and fill the *γ*‐index values for each discrete DLG and transmission value pair as requested by the optimization routine as needed. Cost function F2 was found to be stable against initial start values, therefore was ultimately used (more details on this can be found in the supplemental information).

**FIGURE 2:**
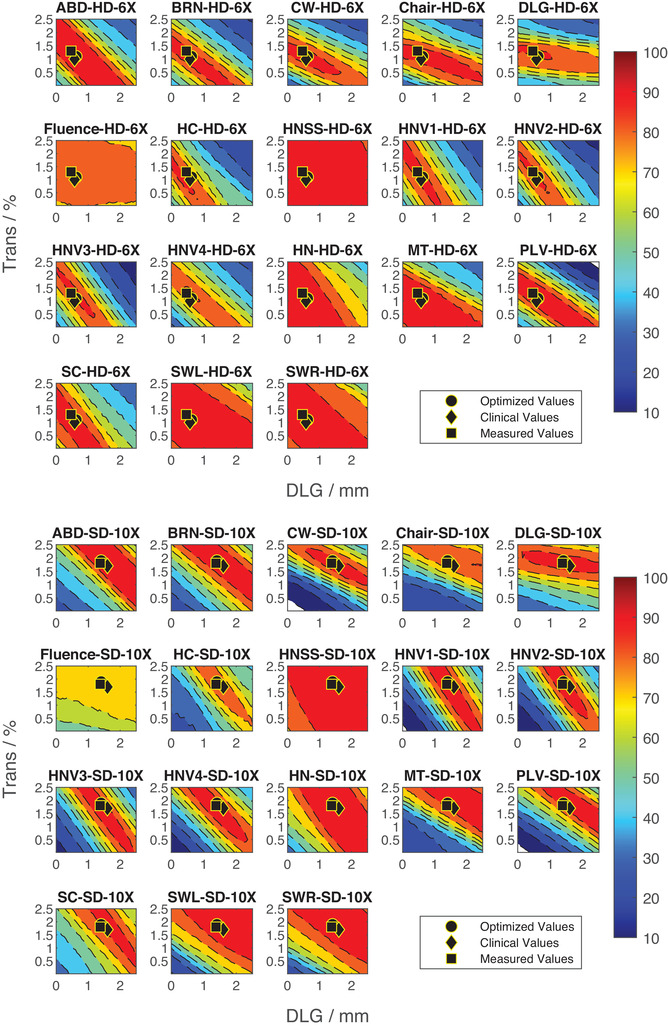
The calculated *γ*‐index pass rates with (2 mm, 2%) criterion as a function of dosimetric leaf gap (DLG) and transmission values for plans with (a) 6X energy and HD‐MLC type and (b) 10X energy and SD‐MLC type. The circle represents the optimized DLG and transmission pair, the diamond represents the current clinical values, and the square represents the measured values. The color scale spans from 10% pass rate (blue) to 100% pass rate (red)

Two examples of the calculated *γ*‐index pass rates for two plans are shown in Figures 2a (6X, HD‐MLC) and 2b (10X, SD‐MLC). In each figure, the pixels represent the *γ*‐index pass rate (color axis), calculated with [2 mm, 2%] criteria, for the corresponding DLG (horizontal axis), and the transmission values (vertical axis). The color scale spans the pass rates in the 10%–100% range.

### 2.5 Validation of automated processes

Prior to automating the processes described here, each step was validated independently using manual techniques. Plans calculated with discretely set DLG and transmission values were compared to those plans calculated using the software developed. The exported dose planes using the scripted approach were compared to dose planes exported using the vendor‐provided functionality of the TPS, and the developed *γ*‐analysis code was verified independently before implementation using test cases.^20^ The results of the comparison of the manual to automated methods for each step of the methods can be viewed in the Supporting Information.

## 3 RESULTS

To validate the optimization, comparisons to the clinically implemented DLG and transmission parameters, as well as to the measured values were performed. Statistical significance was tested using Student's two‐tailed *t*‐test for paired samples. After validating the optimization, the plans held out of the optimization were calculated with the proposed DLG and transmission values and then compared to measurement using *γ*‐analysis. While the optimization utilized the mean *γ*‐index, we present both the *γ*‐index pass rate and mean *γ*‐index for ease of interpretation.

### 3.1 Validation of optimization

**FIGURE 3:**
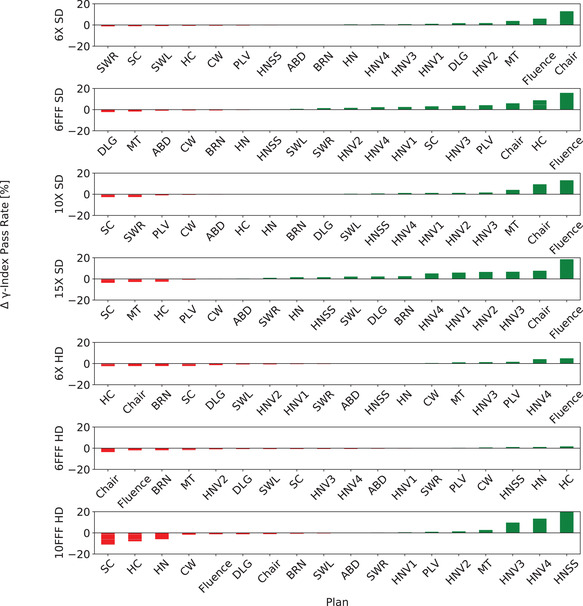
Each pane represents nominal beam energy and multileaf collimator system (MLC) type that displays the difference in *γ*‐index pass rate in percent for each plan used in both optimization and validation (shaded bars) compared with the pass rate calculated with the clinically implemented dosimetric leaf gap (DLG) and transmission values. Green bars indicate a positive difference (better) meaning that the pass rate calculated using the optimized values of DLG and transmission was greater than the pass rates calculated with the clinically implemented values

**FIGURE 4:**
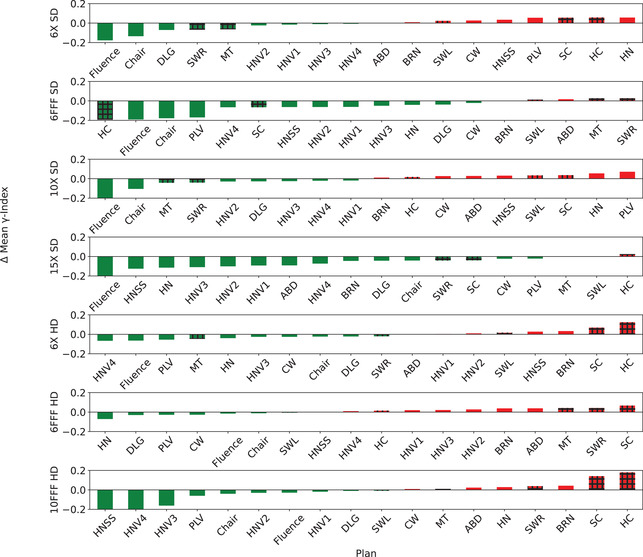
Each pane represents nominal beam energy and multileaf collimator system (MLC) type that displays the difference in mean *γ*‐index for each plan used in both optimization and validation (shaded bars) compared with the mean *γ*‐index calculated with the clinically implemented dosimetric leaf gap (DLG) and transmission values. Green bars indicate a negative difference (better) indicating the mean *γ*‐index calculated using the optimized values of DLG and transmission were less than those calculated with the clinically implemented values

To evaluate the dosimetric differences between the optimized DLG and transmission and clinically used values, the 13 plans used in the optimization and five validation plans were calculated with the optimized values and *γ*‐analysis was performed using [2 mm, 2%] criterion for all energies and MLC types. To visualize the effect of the optimized DLG and transmission value pairs on the mean *γ*‐index and the pass rates for each plan, the change in *γ*‐index parameters was plotted for each energy and MLC type. Figure 3 displays the change in the *γ*‐index pass rate for each energy and MLC type combination and Figure 4 shows the change in mean *γ*‐index for each MLC type and energy pair. The majority of the plans for each energy and MLC type show minor changes (positive and negative) to the *γ*‐index pass rate and mean *γ*‐index. Of note are the combinations of 6X‐FFF and 15X with SD‐MLC, which exhibit a more skewed distribution towards positive changes for *γ*‐index pass rate and negative values for mean *γ*‐index. This is consistent with the results presented in Table 2 showing statistical significance in both pass rate and mean *γ*‐index.

**TABLE 2:** Summary results displaying the γ‐index pass rates and mean γ‐index resulting from evaluation. The dose planes were calculated using dosimetric leaf gap (DLG) and transmission values set to the current clinical values and the optimized values (see Table 3) resulting from our proposed method for each energy and multileaf collimator system (MLC) type. The *p*‐value was calculated using Student's two‐tailed *t*‐test for paired samples with *α* = 0.05 and threshold for statistical significance set to *p* < 0.05

**SD‐MLC**		**TPS**		**Optimized**		**Clinical – optimized**	
**Energy**	**Evaluation**	**Mean**	**Σ**	**Mean**	***σ***		***p*‐value**
6X	Pass rate	0.914	0.087	0.927	0.064	‐0.013	0.125
6X‐FFF	Pass rate	0.880	0.150	0.904	0.121	‐0.024	0.034*
10X	Pass rate	0.920	0.083	0.934	0.051	‐0.015	0.13
15X	Pass rate	0.911	0.096	0.941	0.060	‐0.029	0.028*
6X	Mean gamma	0.454	0.167	0.440	0.115	0.014	0.393
6X‐FFF	Mean gamma	0.533	0.209	0.472	0.158	0.062	0.003*
10X	Mean gamma	0.448	0.148	0.436	0.094	0.012	0.453
15X	Mean gamma	0.470	0.145	0.407	0.127	0.063	0.000*

**HD‐MLC**		**TPS**		**Optimized**		**Clinical – Optimized**	
**Energy**	**Evaluation**	**Mean**	**Σ**	**Mean**	***σ***		***p*‐value**
6X	Pass rate	0.937	0.045	0.937	0.037	0.000	0.965
6X‐FFF	Pass rate	0.947	0.035	0.942	0.036	0.005	0.100
10X‐FFF	Pass rate	0.908	0.076	0.920	0.060	‐0.012	0.530
6X	Mean gamma	0.423	0.081	0.417	0.079	0.006	0.591
6X‐FFF	Mean gamma	0.386	0.077	0.393	0.075	‐0.007	0.372
10X‐FFF	Mean gamma	0.475	0.137	0.444	0.117	0.031	0.360

The results of the optimization were also validated by comparing to the published *γ*‐index pass rates in TG119. Table 3 summarizes these results and shows agreement between the analysis using our method and the published pass rates. While the Hard C plan displayed a pass rate below 90% when analyzed using [2 mm, 2%], the analysis using [3 mm, 3%] shows better agreement than the published values.

**TABLE 3:** The results of the 6X validation plans for both HD and SD‐MLC types as compared to published results of Task Group 119 (TG119) using two different γ‐index criteria: [3 mm, 3%] and [2 mm, 2%]. The published values for TG119 composite plans were analyzed using [3 mm, 3%] for 6X only, and various multileaf collimator system (MLC) types

	**TG119 Published values**				**Measured results**	
**Plan**	**Mean**	**Σ**	**Max**	**Min**	**[3 mm, 3%]**	**[2 mm, 2%]**
Multitarget	99.1	0.9	100	97.5	99.1 ± 0.3	96.3 ± 0.8
Simple C	97.6	3.9	100	87.9	98.4 ± 1.0	92.4 ± 2.6
Hard C	94.4	6.0	99.4	86.2	96.7 ± 1.6	88.5 ± 2.1

### 3.2 Comparison of optimized to TPS and measured values

Table 4 outlines the results of the optimization process as compared to the values that are currently implemented in the TPS identified via an iterative method, which have been validated and are being used clinically. The largest difference between the optimized DLG value and the TPS value is for the HD‐MLC and energy of 6X‐FFF where the optimized DLG value is 0.307 mm and the current Eclipse value is 0.550 mm. The transmission values for all MLC types and energies differed by less than 0.003 (0.3%).

**TABLE 4:** Comparison of optimized dosimetric leaf gap (DLG) and transmission values to those currently in the TPS for all energies and multileaf collimator system (MLC) types

**Energy**	**MLC type**	**Optimized DLG (mm)**	**Optimized trans. (%)**	**TPS DLG (mm)**	**TPS trans. (%)**	**ΔDLG (mm)**	**ΔTrans. (%)**
6X	HD	0.625	1.1	0.575	1.0	0.055	0.1
6X‐FFF	HD	0.459	0.9	0.550	0.8	‐0.091	0.1
10X‐FFF	HD	0.762	1.1	0.530	1.2	0.232	‐0.1
6X	SD	1.030	1.7	1.250	1.4	‐0.220	0.3
6X‐FFF	SD	1.207	1.3	1.200	1.1	0.007	0.2
10X	SD	1.404	1.9	1.600	1.7	‐0.196	0.2
15X	SD	1.429	1.8	1.800	1.6	‐0.371	0.0

Table 5 displays the results of the optimization compared to measured values of DLG and transmission using the technique developed by LoSasso et al.^4^ The largest difference between the measured values and optimized values is for 10X‐FFF with HD‐MLC with a difference of 0.259 mm. The agreement of all transmission values is within 0.002 (0.2%).

**TABLE 5:** Comparison of optimized dosimetric leaf gap (DLG) and transmission values to the measured values using the method described by LoSasso et al.^4^ for all energies and multileaf collimator system (MLC) types

**Energy**	**MLC type**	**Optimized DLG (mm)**	**Optimized trans. (%)**	**Measured DLG (mm)**	**Measured trans. (%)**	**ΔDLG (mm)**	**ΔTrans. (%)**
6X	HD	0.625	1.1	0.463 ± 0.101	1.3 ± 0.1	0.162	‐0.2
6X‐FFF	HD	0.459	0.9	0.383 ± 0.103	1.1 ± 0.1	0.076	‐0.2
10X‐FFF	HD	0.762	1.1	0.503 ± 0.120	1.3 ± 0.0	0.259	‐0.2
6X	SD	1.030	1.7	1.217 ± 0.156	1.5 ± 0.1	‐0.187	0.2
6X‐FFF	SD	1.207	1.3	1.380^a^	1.4^a^	‐0.173	‐0.1
10x	SD	1.404	1.9	1.375 ± 0.179	1.8 ± 0.1	0.029	0.1
15X	SD	1.429	1.8	1.380 ± 0.185	1.7 ± 0.1	0.049	0.1

^a^ There is a single linac with SD‐MLC and 6X‐FFF at our institution.

**FIGURE 5:**
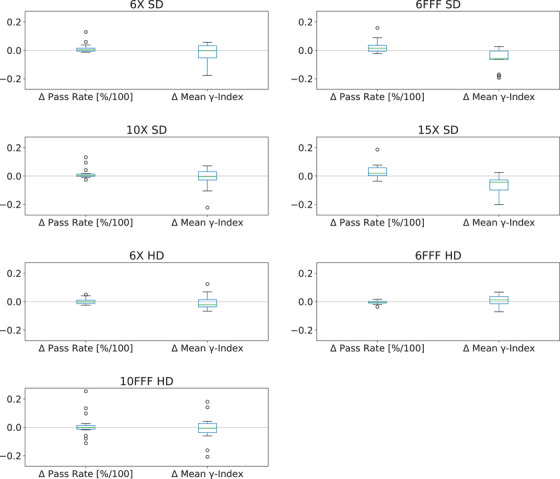
The box and whisker plots show a summary view for the change in both *γ*‐index pass rate and mean *γ*‐index for each nominal beam energy and MLC type pair. For *γ*‐index pass rate, larger values show the optimized values increased the pass rate, and for mean *γ*‐index lower values show better agreement between calculation and measurement than the clinically implemented values of dosimetric leaf gap (DLG) and transmission

A summary of the mean changes for each of the MLC type and energy pairs is visible in Table 6. When compared to the DLG and transmission vales that are clinically implemented, 6/7 energy and MLC type pairs showed improvement by way of an increase of *γ*‐index pass rate and decrease of mean *γ*‐index. Figure 5 shows the summary results of mean change for each MLC type and energy pair using a box and whisker plot. The results for the 15X SD pair show the magnitude of improvement implementation of the optimized values would make with regards to the *γ*‐index pass rate and mean *γ*‐index.

**TABLE 6:** The mean change in γ‐index pass rate and mean γ‐index for each energy and multileaf collimator system (MLC) type pair along with the range of those changes are displayed here. The plans were compared with (2%, 2 mm), 10% threshold, and global normalization γ‐index criteria. When compared to the dosimetric leaf gap (DLG) and transmission parameters clinically implemented, higher pass rates and lower mean γ‐index show improvement. The final column shows the percentage of plans that showed improvement by application of the optimized DLG and transmission parameters

**Energy**	**MLC type**	$\bar{\Delta }$ **Pass Rate (±σ)**	**Range [%]**	**Percent of plans with improvement**
6X	HD	0.02 ± 2.09%	(‐2.45, 4.96)	38.9%
6X‐FFF	HD	‐0.54 ± 1.31%	(‐3.64, 1.74)	33.3%
10X‐FFF	HD	1.23 ± 8.17%	(‐11.16, 25.38)	44.4%
6X	SD	1.31 ± 3.44%	(‐1.32, 12.94)	55.6%
6X‐FFF	SD	2.39 ± 4.40%	(‐2.32, 15.83)	61.1%
10X	SD	1.48 ± 3.94%	(‐2.65, 13.18)	66.7%
15X	SD	2.93 ± 5.16%	(‐3.65, 18.58)	77.8%

**Energy**	**MLC type**	$\bar{\Delta }$ **Mean γ‐index (±σ)**	**Range**	**Percent of plans with improvement**
6X	HD	‐0.01 ± 0.05	(‐0.07, 0.12)	61.1%
6X‐FFF	HD	0.01 ± 0.03	(‐0.07, 0.07)	38.9%
10X‐FFF	HD	‐0.03 ± 0.14	(‐0.46, 0.18)	55.6%
6X	SD	‐0.01 ± 0.07	(‐0.18, 0.06)	50.0%
6X‐FFF	SD	‐0.06 ± 0.07	(‐0.19, 0.03)	72.2%
10X	SD	‐0.01 ± 0.07	(‐0.22, 0.07)	50.0%
15X	SD	‐0.06 ± 0.06	(‐0.20, 0.02)	83.3%

## 4 DISCUSSION

In this study, we have presented a methodology whereby a phase space was precalculated for clinically relevant DLG and transmission values for two types of MLC and a variety of nominal beam energies. The unaltered treatment plans were delivered, measured, and analyzed via commonly employed techniques. The optimal values of DLG and transmission were located by minimizing a cost function constructed of the mean *γ*‐index. Optimality was tested by running the optimization with systematic variation in the starting search parameters, by which, the mean *γ*‐index was found to be stable regardless of starting search parameters. Moreover, our results confirm previous studies’ findings regarding the plan‐dependent nature of the DLG and transmission optimal pairs and discover a new challenge when attempting to use *γ*‐index for this process.^5^, ^13^

With the exception of 6X‐FFF and 15X energies of SD‐MLC, no statistically significant differences between the clinically used values and the optimized values were found. The statistically significant changes in *γ*‐index pass rate and mean *γ*‐index for the SD‐MLC 15X energy can be attributed to our institution not performing IMRT or VMAT with 15X, thus a loosening of the tolerance used when evaluating DLG and transmission during commissioning. The statistically significant change in pass rate and mean *γ*‐index for SD‐MLC 6X‐FFF highlights the challenges of using the iterative method for tuning DLG and transmission during commissioning. The disagreement between the clinically implemented values and optimized values has served as an impetus for further investigation during an upcoming TPS upgrade. Figure 2 shows clear evidence of a non‐trivial relationship between DLG and transmission within a single plan, but also across multiple plans within the same energy and MLC type. Further, by using *γ*‐index features, a unique optimal solution is unattainable as confirmed by previous work.^5^ Multiple solutions exist where the mean *γ*‐index and the *γ*‐index pass rates are equal. When utilizing an iterative method to adjust DLG and transmission, this may act as a benefit by which small adjustments do not measurably change each iteration, but when determining optimality, new metrics for comparison should be sought after.

Further reviewing Figure 2, each plan has an area of high agreement for many DLG and transmission values. Table 6 shows the percentage of plans for each energy and MLC type that improved as a result of applying the optimized DLG and transmission values. Three of the energy and MLC pair combinations show less than 50% of plans had an improvement in the pass rate. The relatively large areas of high *γ*‐index pass rates are the most probable cause. Further, *γ*‐index pass rate and mean *γ*‐index are not directly proportional. Optimization on mean *γ*‐index will not directly result in an increase in the *γ*‐index pass rate.

This method will reduce the amount of time required for physicists to perform the tuning of the DLG and transmission parameters as well as reduce the probability that non‐optimal parameters are chosen. The measurements required are identical to the iterative approach often used. Furthermore, the library of plans generated can be used to simplify quality assurance of the MLCs post‐service, post‐upgrade, or on a routine basis. For instance, a physicist may measure the plans after service and perform the optimization to ensure the values for DLG and transmission are within a specified tolerance. This is contrary to measuring the DLG and transmission values explicitly to determine if the change has occurred. As shown here, the measured DLG and transmission values do not correspond to the optimal values. As a result, it is difficult to ascertain the magnitude of change in the measured values that would necessitate intervention.

This study has some limitations. The number of plans utilized during the optimization is relatively small. Increasing the number of plans would provide more parameters for the optimization to ensure clinical applicability to a larger variety of treatment plans. Along with the number of plans, the current technique is naïve. Each plan is valued equally in the optimization although one may provide less information than another. For instance, it is evident that step‐and‐shoot IMRT plans provide little benefit as evidenced by the consistency of pass rate (see HNSS in Figure 3). As well as the number of plans, the types of plans used are a limitation. However, we see the inclusion of more diverse group plans and VMAT delivery as an extension of this work.

## 5 CONCLUSION

The methodology presented in this work identifies the optimal values of DLG and transmission using the calculation of phase space for clinical treatment plans. The automated methodology uses the mean *γ*‐index as the cost function during optimization, which proved to be stable and robust against starting conditions of the optimization. Additionally, it was validated for plans excluded from the optimization and displayed agreement with clinically implemented DLG and transmission values in the TPS, identified via an iterative approach. This method will reduce the time required to determine the clinically acceptable parameters and can ensure the optimality of DLG and transmission for the plans included in the optimization.

## AUTHOR CONTRIBUTIONS

Both authors equally contributed to the conceptual design of the work, acquisition, analysis, and interpretation of the data, drafting and revising the manuscript; will jointly provide the final approval of the version to be published, and agree to be accountable for all aspects of this work.

## Supporting information

Supporting Information

Supporting Information

Supporting Information

Supporting Information

Supporting Information

Supporting Information

Supporting Information

Supporting Information

Supporting Information
