# Supplementary material for: A novel methodology for the optimization of transmission and dosimetric leaf gap parameters
Source: J Appl Clin Med Phys. 2022 Feb 28;23(5):e13565. doi: 10.1002/acm2.13565 (PMC9121040; doi:10.1002/acm2.13565)
Supplement: Supplementary file 9 — Supporting Information [file ACM2-23-e13565-s005.docx]

# Supplemental Information

## Validation of Scripted TPS Workflow

To validate the effect of our methods as compared with the manual TPS workflow, we devised three comparisons. The plan HNV2 for nominal beam energy 6X-FFF and HD MLC type is shown as an example throughout the supplemental information. The first comparison utilized the standard workflow in the TPS to generate a ground truth to compare against. The procedure to validate the scripted calculation and export follow.

The DLG was set to 1.25mm and transmission to 0.0125 in RT Administration of the TPS. The source plan with unaltered MLC positions was calculated and then the isocenter dose plane was exported using the TPS functionality. Next, the DLG parameter was set to 0.001mm while the transmission value remained at 0.0125. The script developed for this work was used to calculate the plan with the virtual DLG of 1.25mm by moving each MLC by 0.625mm. The script exported a dose plane at isocenter after the calculation was completed, and then saved the information in the TPS. The plan was opened in the TPS and the isocenter dose plane was exported manually using the TPS functionality. Three dose planes now existed for comparison: (1) TPS calculated and TPS exported, (2) script calculated and TPS exported, and (3) script calculated and script exported. All dose planes exported, whether by script or TPS, had a pixel spacing of 1mm and were 400mm in both the x and y directions. The resulting comparisons can be viewed in Figures S1, S2, and S3.

Figure S1 compares the TPS calculated and exported dose plane to the script calculated and TPS exported dose plane. Minor differences exist between the two as shown by the light gray dashed line on the secondary y-axis. The maximum difference was -12.65cGy, with a mean absolute difference of 0.45cGy. Of the total pixels in the dose plane, 160,801, only 2.22% (3,577 pixels) differed by more than 1%. This shows that the methodology for implementing the DLG by physically adjusting individual MLC positions is a reasonable alternative to manually adjusting the DLG parameter in the TPS. Since the export was performed utilizing the same methodology, any differences visible between the two dose planes must be due to differences in manually setting the DLG in the TPS and the scripted method.

Figure S2 compares the scripted method of DLG implementation exported via the TPS functionality to the scripted method of DLG implementation exported via the scripted method. Again, minor differences can be seen between the two dose planes. The maximum difference between the two planes was -10.70cGy with 0.74% of all pixels exceeding a difference of 1%. This comparison shows that the differences between the TPS functionality to export a dose plane the scripted method are nearly identical.

Figure S3 compares a dose plane generated using manual techniques in the TPS for setting the DLG parameter and exporting dose planes to the fully automated method. In this case, minor differences can be seen, as expected based on the previous two comparisons. The maximum difference visible in this comparison was 14.89cGy with 3.04% of all pixels exceeding a 1% absolute dose difference. Based on these comparisons, the scripted method developed to automatically adjust the DLG by physically moving the MLC positions, and then export the dose planes can be considered a reasonable surrogate to the manual processes.

## Validation of *γ*-index Calculations

Two of the test distributions provided in the work by Agnew and McGarry^20^ are geometric constructs. In the first test dose distribution, the distance-to-agreement (DTA) is validated, while in the second dose distribution the percent dose difference (%DD) is validated. The theoretically expected *γ*-index pass-rates were compared against the pass-rates calculated by the developed code for both test dose distributions. In both cases, the pass-rates for %DD and DTA analysis were found to be identical to the expected values.

The *γ*-index was calculated using an in-house developed program written in MATLAB. The TPS calculated dose planes were taken as the *Reference* dose distribution, and the MapCHECK measured dose distributions were assigned as the *Evaluated*. Similar to our clinical practice, the *γ*-index calculations were performed for the *Evaluated* measurement points which are above the 10% of the maximum dose.

As stated in the manuscript, the developed code was bench marked using the *Reference* and *Evaluated* dose distributions provided in the Agnew and McGarry.[20](#_bookmark19) Since these dose distributions are geometric constructs, their theoretical pass rates could be calculated. Our developed *γ*-index calculation code matched the theoretically expected pass rates exactly. Figures [S4](#_bookmark22) and [S5](#_bookmark23) show the two example dose distributions, the "DTA" and "Dose Difference", used for *γ*-index calculation code validation.

## Stability of Optimization of DLG and Transmission Parameters with Cost Functions

Two cost functions were evaluated: the sum of the pass rates (F1) and the sum of the mean of *γ*-index (F2) values for each evaluated plan

$$F1=max\left\{ \sum_{i=1}^{allplans} \left[ \Gamma_{PassRate} \right]_{i} \right\}$$

and

$$F2=min\left\{ \sum_{i=1}^{allplans} \bar{\Gamma}_{i} \right\}.$$

The search for the optimal DLG and transmission value pairs by *fmincon* for each cost function was repeated with varying initial starting points. In Figures [S](#_bookmark24)6 and [S](#_bookmark25)7, the converged DLG and transmission pair values are shown for all optimization starting points. Figure [S6](#_bookmark24) shows primarily two convergence locations for all plans with different MLC types and energies when pass rate metric (F1) was used. The mean *γ*-index based (F2) optimization (see Figure [S7](#_bookmark25)) does not exhibit this behavior. Note the difference in the DLG and transmission axis scales of the corresponding histograms in both figures.

Figure S8 shows the relationship between the calculated *γ*-index pass rate and mean *γ*-index values for all plans and DLG transmission pairs. It is observed that mean *γ*-index values of < 1.5 provide better selectivity. A small change in *γ*-index pass rate, corresponds to a larger change in the mean *γ*-index.

Figure Captions

Figure S1: Comparison of dose planes from the plan HNV2 for the HD MLC and 6X-FFF nominal beam energy using two separate techniques. *TPS Calculated and Exported* image shows a dose plane generated by setting the DLG (1.25mm) and transmission (0.0125) in RT Administration for the specified energy. The pane *Script Calculated and TPS Exported* shows the surrogate method used in this study to move the MLC positions in lieu of setting the DLG in RT Administration.

Figure S2: Comparison of dose planes from the plan HNV2 for the HD MLC and 6X-FFF nominal beam energy using two separate techniques. *TPS Exported* image shows a dose plane generated by physically moving the MLC positions but then exported using in-built TPS functionality. The pane *Script Calculated and Exported* shows same method of setting the DLG, but the export was performed using a scripted process.

Figure S3: Comparison of dose planes from the plan HNV2 for the HD MLC and 6X-FFF nominal beam energy using two separate techniques. *TPS Calculated and Exported* image shows a dose plane generated by setting the DLG (1.25mm) and transmission (0.0125) in RT Administration for the specified energy. The pane *Script Calculated and Exported* shows the automated method for setting the DLG and export process.

Figure S4: Validation of the in-house developed *γ*-index calculation program for “DTA test distributions” from Agnew and McGarry.^20^

Figure S5: Validation of the in-house developed *γ*-index calculation program for “dose difference test distributions” from Agnew and McGarry.^20^

Figure S6: Converged DLG and transmission pair values with optimization with *γ*-index pass rate (F1).

Figure S7: Converged DLG and transmission pair values with optimization with mean *γ*-index (F2).

Figure S8: The relationship between the mean gamma index and the corresponding gamma index is shown for all DLG and transmission pairs and plans.
